# Overexpression of *HMGA1* Figures as a Potential Prognostic Factor in Endometrioid Endometrial Carcinoma (EEC)

**DOI:** 10.3390/genes10050372

**Published:** 2019-05-15

**Authors:** Antonio Palumbo Júnior, Vanessa Paiva Leite de Sousa, Francesco Esposito, Marco De Martino, Floriana Forzati, Fábio Carvalho de Barros Moreira, Tatiana de Almeida Simão, Luiz Eurico Nasciutti, Alfredo Fusco, Luis Felipe Ribeiro Pinto, Cláudia Bessa Pereira Chaves, Nathalia Meireles Da Costa

**Affiliations:** 1 Programa de Carcinogênese Molecular, Instituto Nacional de Câncer—INCA, Rua André Cavalcanti, 37 - Centro, Rio de Janeiro, RJ 20231-050, Brazil; palumbo@icb.ufrj.br (A.P.J.); vpl_sousa@hotmail.com (V.P.L.d.S.); tasimao@gmail.com (T.d.A.S.); alfusco@unina.it (A.F.); lfrpinto@inca.gov.br (L.F.R.P.); claudia.bessa67@gmail.com (C.B.P.C.); 2 Laboratório de Interações Celulares, Instituto de Ciências Biomédicas, Universidade Federal do Rio de Janeiro Prédio de Ciências da Saúde—Cidade Universitária, Ilha do Fundão, A. Carlos Chagas, 373—bloco F, sala 26, Rio de Janeiro, RJ 21941-902, Brasil; luiz.nasciutti@histo.ufrj.br; 3 Istituto di Endocrinologia e Oncologia Sperimentale—CNR c/o Dipartimento di Medicina Molecolare e Biotecnologie Mediche, Università degli Studi di Napoli “Federico II”, via Pansini 5, 80131 Naples, Italy; francesco.esposito2@unina.it (F.E.); marco.demartino2@unina.it (M.D.M.); floriana.forzati@gmail.com (F.F.); 4 Divisão de Patologia, Instituto Nacional de Câncer—INCA, Rua Cordeiro da Graça, 156—Santo Cristo, Rio de Janeiro, RJ 20220-040, Brazil; fcbmoreira@hotmail.com; 5 Laboratório de Toxicologia e Biologia Molecular, Departamento de Bioquímica, Instituto de Biologia Roberto Alcântara Gomes, Universidade do Estado do Rio de Janeiro, Av. 28 de setembro, 87—fundos—4º andar, Rio de Janeiro, RJ 20551-030, Brazil; 6 Seção de Ginecologia Oncológica, Hospital de Câncer II, Instituto Nacional de Câncer—INCA, Rua Equador, 835. Santo Cristo, Rio de Janeiro, RJ 20220-410, Brazil

**Keywords:** endometrial carcinomas, cancer progression, molecular biomarkers

## Abstract

Endometrioid endometrial carcinomas (EEC) are the most common malignant gynecologic tumors. Despite the increase in EEC molecular knowledge, the identification of new biomarkers involved in disease’s development and/or progression would represent an improvement in its course. High-mobility group A protein (HMGA) family members are frequently overexpressed in a wide range of malignancies, correlating with a poor prognosis. Thus, the aim of this study was to analyze HMGA1 and HMGA2 expression pattern and their potential role as EEC biomarkers. HMGA1 and HMGA2 expression was initially evaluated in a series of 46 EEC tumors (stages IA to IV), and the findings were then validated in The Cancer Genome Atlas (TCGA) EEC cohort, comprising 381 EEC tumors (stages IA to IV). Our results reveal that HMGA1 and HMGA2 mRNA and protein are overexpressed in ECC, but only *HMGA1* expression is associated with increased histological grade and tumor size. Moreover, *HMGA1* but not *HMGA2* overexpression was identified as a negative prognostic factor to EEC patients. Finally, a positive correlation between expression of *HMGA1* pseudogenes—*HMGA1-P6* and *HMGA1-P7*—and HMGA1 itself was detected, suggesting HMGA1 pseudogenes may play a role in HMGA1 expression regulation in EEC. Thus, these results indicate that *HMGA1* overexpression possesses a potential role as a prognostic biomarker for EEC.

## 1. Introduction

Endometrial carcinomas (ECs) are the most common malignant gynecologic tumors, with an estimated 320,000 new cases and 76,000 related deaths expected per year [1]. Etiological factors such as obesity, persistent anovulatory cycles, nulliparity and exogenous estrogen exposure are intrinsically associated with the malignancy. The main EC treatment is surgery, and adjuvant therapy can also be applied, depending on specific tumor behaviors, such as myometrial invasion [2,3,4]. Importantly, myometrial invasion rates increase along with tumor staging and represent an independent predictive outcome factor. Deep invasion is frequently associated with poor-differentiated tumors, lymph node metastasis, high recurrence rates and decreased overall survival [5,6]. Adenocarcinomas represent about 90% of EC cases and are subdivided in to type I or II according to both histological characteristics and clinical behavior [7]. Type I adenocarcinomas represent approximately 85% of cases and are known as endometrioid endometrial tumors (EEC). These tumors arise from previous hyperplasia, are estrogen-dependent, well- to moderately-differentiated, related to obesity and generally present good prognosis, with most of them being surgically curable [8]. Despite the increasing knowledge in molecular alterations present in EC, many questions remain unanswered. Enhanced effort in the identification of molecular markers involved in the development of the disease or its prognosis would represent an improvement in disease course [9].

The high-mobility group A proteins (HMGAs) are a family of small non-histone chromatin factors, encoded by the genes *HMGA1* and *HMGA2*, which play a role in malignant cell transformation and progression of different tumors [10,11,12,13,14,15,16,17]. HMGAs proteins are involved in gene transcription regulation, acting through either the enhancement or suppression of transcription factors’ activity by remodeling chromatin structure and orchestrating transcription factors multiprotein complexes recruitment [18,19]. While *HMGA* genes are expressed at very low levels in normal adult tissues, they are frequently overexpressed in a wide range of tumors, commonly predicting poor prognosis [20]. The mechanisms by which HMGA proteins are involved in cell transformation are mainly based on their capacity of modulating the expression of genes involved in cell proliferation and invasion control [20].

HMGA proteins have already been reported to be involved in development of female genital tract tumors. HMGA1-induced expression targeted to mice uterine tissues is capable of driving the development of uterine adenosarcoma [21]. Additionally, high levels of *HMGA1* mRNA were detected in high-grade, aggressive uterine tumors when compared with less aggressive uterine neoplasms [22]. The involvement of HMGA2 in uterine neoplastic transformations is less clear. Nevertheless, increased levels of HMGA2 are only found in aggressive, invasive endometrium carcinomas [23].

Even though HMGA upregulation in tumors and its role in malignant cell transformation are well identified, their mRNA and protein levels regulation has not been comprehensively elucidated yet. Recent studies have demonstrated the involvement of epigenetic mechanisms, represented by non-coding RNAs, in both *HMGA1* and *HMGA2* regulation. For instance, two *HMGA1* pseudogenes have been recently described, *HMGA1P6* and *HMGA1P7.* These pseudogenes regulate HMGA1 protein levels by preventing their degradation mediated by miRNAs and possessing oncogenic characteristics [24,25].

In order to assess whether HMGA1 and HMGA2 may serve as prognostic factors in EEC, we evaluated their mRNA and protein expression profile in EEC tumors ranging from stage IA to stage IV and correlated the clinicopathological features with the molecular findings. Additionally, we investigated *HMGA1-P6* and *HMGA1-P7* pseudogenes expression in order to verify whether they would play a role in *HMGA* genes expression regulation.

## 2. Material and Methods

### 2.1. Patients and Samples

Forty-six patients with confirmed histological diagnosis of endometrioid endometrial carcinoma (EEC), who underwent surgical treatment between 2007 and 2009 at INCA (Brazilian National Cancer Institute, Rio de Janeiro, Brazil) and did not undergo chemo/radiotherapy, were included. Histological diagnosis was confirmed by two independent pathologists after surgical treatment. Six patients, who underwent total hysterectomy due to any clinical reason other than endometrial cancer in the same period, were also included (five atrophic and one proliferative endometrium samples). These patients, included in the study as controls for EEC development, did not undergo chemo/radiotherapy either and possessed clinicopathological characteristics similar/comparable to those of the EEC patients. Tumor size was measured by assessing the postoperative surgical specimen. Epidemiological and clinicopathological data were obtained through interviews by using a standardized questionnaire and from patients’ medical records, respectively. Tumor and normal endometrial samples were formalin-fixed paraffin-embedded (FFPE). The protocol was approved by the Institutional Ethics Committee, and all patients signed a consent form.

### 2.2. RNA Extraction, Reverse Transcription and RT-qPCR

Total RNA was extracted from EEC and normal endometrial epithelium FFPE samples using PureLink^TM^ FFPE Total RNA Isolation Kit (Invitrogen^®^) according to manufacturer’s instructions. All RNA samples were measured by spectrophotometry, and cDNA was synthesized from 1 μg of RNA by using the SuperScript^TM^ II Reverse Transcriptase (Invitrogen^®^) protocol, and then real-time quantitative PCR (RT-qPCR) was performed by using SYBR Green Master Mix (Qiagen) and specific primers for the genes investigated. Primer sequences can be found on Table S1. Differential gene expression was calculated as described elsewhere [26].

### 2.3. Immunohistochemistry

Immunohistochemistry (IHC) was performed on 3 μm paraffin sections of 20 EEC cases. HMGA1 (Abcam AB129153, working dilution 1:500) and HMGA2 (Abcam, AB52039, working dilution 1:50) immunostaining was performed as previously described [27,28]. The staining score evaluation was performed by a pathologist, blinded for clinicopathological parameters. For both proteins, scored cases were considered 1+ when positive staining was present in up to 25% of tumor region, 2+ when staining was present in >26% and ≤50% of tumor region, 3+ when present in >51% and ≤75% of tumor region and 4+ when >76% of tumor region was positive. Anaplastic thyroid carcinoma samples were used as positive controls for HMGA1 and HMGA2 staining. As negative control, the primary antibody was replaced by the diluent solution.

### 2.4. Analyses of HMGA1 and HMGA2 Expression Data Deposited in the Cancer Genome Atlas (TCGA)

*HMGA1* and *HMGA2* expression data from 381 EEC samples and 20 endometrial histologically normal tumor-surrounding tissue as well as epidemiological and clinicopathological patients’ data were downloaded from TCGA database.

### 2.5. Statistical Analysis

Frequencies of clinicopathological data and *HMGA1* and *HMGA2* mRNA expression levels were calculated. For continuous variables, descriptive analysis of central and dispersion tendencies was performed. To assess the relationship between mRNA expression levels and clinicopathological features, Fisher’s exact, ANOVA or Kruskal–Wallis, and *t*-test or Mann–Whitney tests were used according to Gaussian distribution. For the correlation analyses we used Pearson’s *r* or Spearman’s rho tests. The statistical analyses and ROC curve were performed with GraphPad Prism 5.0 (GraphPad Software Incorporated, San Diego, CA, USA). The final values were considered of statistical significance when *p* < 0.05. Survival analyses were estimated by the Kaplan–Meier method and log-rank test, based on a confidence interval of 95%. Data with *p* < 0.05 were considered statistically significant. Variables with *p* < 0.2 were selected for multivariate analysis. Finally, Cox regression was applied with the stepwise forward method. Survival analyses were performed by using R [29].

### 2.6. Ethics Approval and Consent to Participate

The use of the human samples was approved by the Ethics Committee of the Brazilian National Cancer Institute (INCA) (approbation number 091/2010, on the 28^th^ February 2011). All patients and healthy individuals, who kindly agreed to participate in the study, signed a consent form and authorized the scientific divulgation of the results obtained from the human samples donated.

## 3. Results

### 3.1. Clinicopathological Features

Table 1 shows EEC patients’ clinicopathological characteristics. Patients’ overall survival was 67.4% in a follow-up period of 108 months (median overall survival time of 66.7 months). Relapse was observed in 28.3% of the cases and, among them, 75.0% died of cancer. The median age of patients was 64 years, ranging from 42 to 83 years. Most of the patients presented family history of cancer (47.82%), hypertension (63.0%) and obesity or overweightness (76.1%). The clinical characteristics of the six control women evaluated in the study are also illustrated in Table 1, showing that the only feature significantly different between cases and controls was their age. Association of overall survival and disease-free survival with all EEC patients’ clinicopathological data was performed, and a significant association between tumor staging and overall survival (*p* = 0.043; HR = 2.97) and disease-free survival (*p* = 0.006; HR = 6.93) was observed (Figure S1A,B).

### 3.2. HMGA1 is Overexpressed in EEC and Its Expression Positively Correlates with Increasing Tumor Staging, Grade and Size

**Figure 1:**
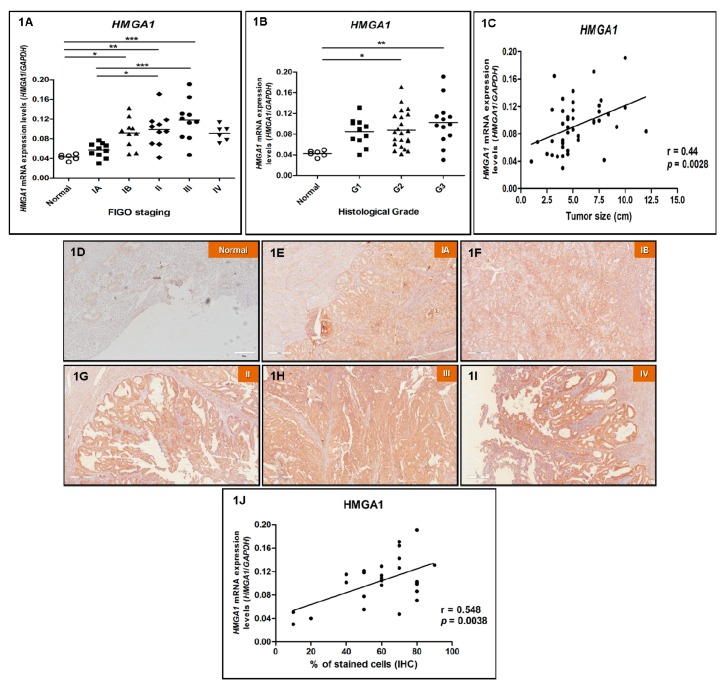
High-mobility group A protein 1 (HMGA1) mRNA and protein expression pattern in endometrioid endometrial adenocarcinomas (EEC). HMGA1 gene and protein expression analysis of the endometrioid endometrial adenocarcinoma (EEC) patients analyzed in this study, according to clinicopathological characteristics, by real-time quantitative PCR (RT-qPCR) and immunohistochemistry (IHC), respectively. (**A**) Tumor staging: *HMGA1* mRNA expression performed in 10 EEC samples from stage IA, 10 from stage IB, 10 from stage II, 10 from stage III, six samples from stage IV and in six normal endometrial samples. (**B**) Histological grade: *HMGA1* mRNA expression performed in 11 well differentiated (G1), 22 moderately differentiated (G2), 13 poorly differentiated (G3) EEC samples and in six normal endometrial samples. (**C**) Tumor size: Correlation between *HMGA1* mRNA levels and tumor size. Evaluation of HMGA1 protein expression was performed in six normal endometrial tissue samples (**D**), five EEC samples from stage IA (**E**), five from stage IB (**F**), five from stage II (**G**), five from stage III (**H**) and five samples from stage IV (**I**) chosen randomly out of the 46 patients analyzed for gene expression. Magnification of photomicrographs: 100×. (**J**) Correlation between *HMGA1* mRNA and protein expression. Statistical analyses were performed based on a confidence interval of 95%. Data with *p* < 0.05 were considered statistically significant. * = *p* < 0.05; ** = *p* < 0.005; *** = *p* < 0.0005.

First, we evaluated *HMGA1* mRNA levels in 10 EEC samples from stage IA, 10 from stage IB, 10 from stage II, 10 from stage III, six samples from stage IV and in six normal, non-cancerous endometrial samples by RT-qPCR. There was a significant increase in the expression of *HMGA1* in IB, II and III stage tumors, compared with normal endometrial samples. The increase in mRNA median expression values was of 1.3-, 2.2-, 2.3-, 2.7- and 2.1-fold in tumor Stage IA, IB, II, III and IV groups, respectively, when compared with control group (normal endometrium). A higher expression of *HMGA1* mRNA levels in II and III tumor stages was also observed when comparing with IA stage tumor (*p* = 0.0001) (Figure 1A). Considering the histological grade of tumors, a statistically significant augmentation of *HMGA1* expression was found in moderately and poorly differentiated tumors (G2 and G3, respectively) when compared with normal endometrial samples (Figure 1B). Furthermore, data analysis indicated a positive correlation between the increase of *HMGA1* gene expression and tumors size (r = 0.44, *p* = 0.0028) (Figure 1C). A statistically significant association was also detected between *HMGA1* expression levels and depth of myometrial invasion. Nevertheless, no significant association between *HMGA1* gene expression and any other clinicopathological feature (Table S2) nor age (r = −0.09819, *p* = 0.5162, data not shown).

Next, aiming to verify whether HMGA1 protein expression follows the same pattern observed for mRNA levels, IHC was performed in the same six normal, non-cancerous endometrial tissues; five EEC samples from stage IA; five from stage IB; five from stage II; five from stage III and six samples from stage IV analyzed for gene expression. The 26 EEC samples assessed by immunohistochemistry were chosen randomly out of the total samples investigated for mRNA expression. As observed for gene expression, all samples were positive for HMGA1 staining and its intensity was higher in EEC samples from stages IB, II, III and IV when compared with samples from stage IA and normal endometrial samples (Figure 1D,I,J and Table S3). In addition, a significant positive correlation between gene and protein expression was observed (r = 0.548; *p* = 0.0038) (Figure 1).

### 3.3. HMGA1P6 and HMGA1P7 Expression Follows the Same Pattern Observed for HMGA1 Expression

**Figure 2:**
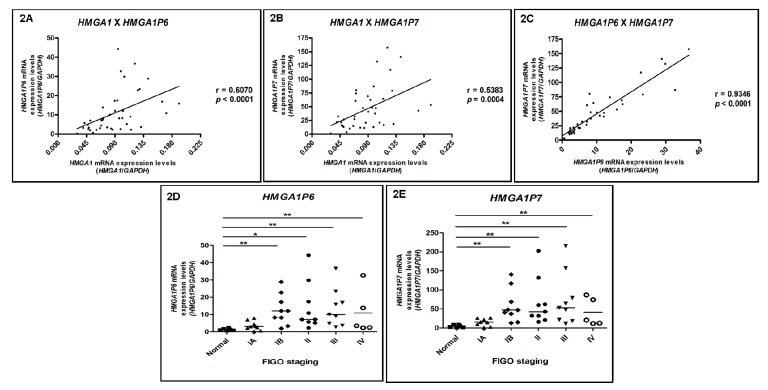
*HMGA1-P6* and *HMGA1-P7* mRNA expression pattern in endometrioid endometrial adenocarcinomas (EEC). Correlation of *HMGA1* mRNA expression with those of *HMGA1P6* and *HMGA1P7* and evaluation of their expression, according to clinicopathological characteristics, by qRT-PCR. *HMGA1P6* and *HMGA1P7* gene expression was performed in six normal endometrial samples, 10 EEC samples from stage IA, 10 from stage IB, 10 from stage II, 10 from stage III and six samples from stage IV, and the (**A**) correlation between *HMGA1* and *HMGA1P6*, (**B**) *HMGA1* and *HMGA1P7* and (**C**) *HMGA1P6* and *HMGA1P7* mRNA levels was evaluated. (**D**) *HMGA1P6* and (**E**) *HMGA1P7* mRNA expression distribution in the different tumor staging groups analyzed. Statistical analyses were performed based on a confidence interval of 95%. Data with *p* < 0.05 were considered statistically significant. * = *p* < 0.05.

Aiming to define whether *HMGA1P6* and *HMGA1P7* participate in *HMGA1* expression regulation, their mRNA levels were investigated in the same EEC series (10 EEC samples from stage IA, 10 from stage IB, 10 from stage II, 10 from stage III, six samples from stage IV) and in six normal endometrial samples by RT-qPCR. A significant positive correlation was observed between *HMGA1* and *HMGA1P6* (r = 0.6070; *p* < 0.0001) (Figure 2A), *HMGA1* and *HMGA1P7* (r = 0.5383; *p* = 0.0004) (Figure 2B) and *HMGA1P6* versus *HMGAP7* expression levels (r = 0.9346; *p* < 0.0001) (Figure 2C). Additionally, a significant increase in *HMGA1P6* and *HMGA1P7* expression was observed along tumor staging augmentation. For *HMGA1P6*, a 2.45-, 15.09-, 5.72-, 7.98-, and 9.15-fold increase was detected in mRNA median expression values of tumor stages IA, IB, II, III and IV, respectively, when compared with normal endometrial samples’ median expression value (Figure 2D). Similarly, for *HMGA1P7*, the augmentation in mRNA median expression values observed was of 3.19-, 9.79-, 8.82-, 10.94- and 6.28-fold in tumor stages IA, IB, II, III and IV, respectively, when compared with normal endometrial samples’ median expression value (Figure 2E).

### 3.4. HMGA2 Expression Does Not Correlate with Increasing Tumor Staging and Tumor Size in EEC

**Figure 3:**
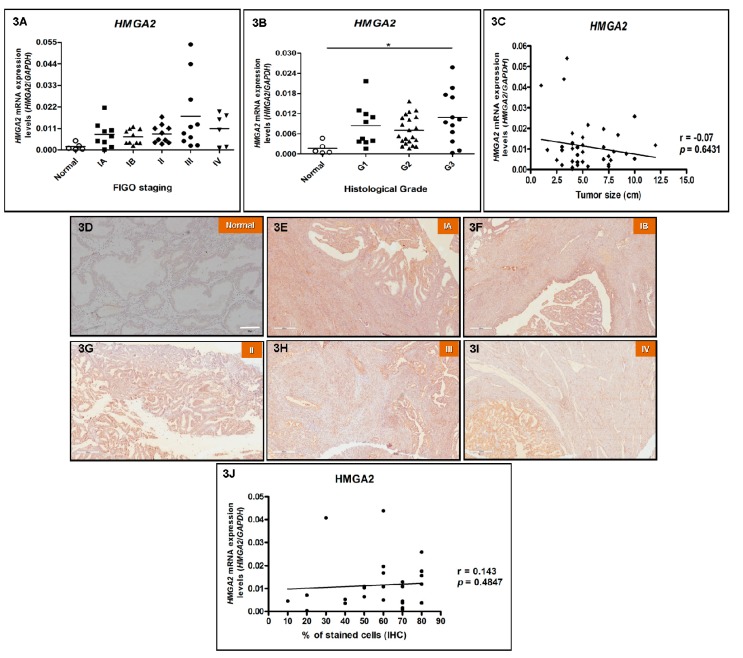
HMGA2 mRNA and protein expression pattern in endometrioid endometrial adenocarcinomas (EEC). *HMGA2* gene expression analysis of the endometrioid endometrial adenocarcinoma (EEC) patients analyzed in this study, according to clinicopathological characteristics, by RT-qPCR and immunohistochemistry (IHC), respectively. (**A**) Tumor staging: *HMGA2* mRNA expression performed in 10 EEC samples from stage IA, 10 from stage IB, 10 from stage II, 10 from stage III, six samples from stage IV and in six normal endometrial samples. (**B**) Histological grade: *HMGA1* mRNA expression performed in 11 well differentiated (G1), 22 moderately differentiated (G2), 13 poorly differentiated (G3) EEC samples and in six normal endometrial samples. (**C**) Tumor size: Correlation between *HMGA2* mRNA levels and tumor size. HMGA2 protein expression analysis in 25 endometrioid endometrial adenocarcinoma (EEC) patients by immunohistochemistry (IHC). Evaluation of HMGA2 protein expression was performed in six normal endometrial tissue samples (**D**), five EEC samples from stage IA (**E**), five from stage IB (**F**), five from stage II (**G**), five from stage III (**H**) and five samples from stage IV (**I**) chosen randomly out of the 46 patients analyzed for gene expression. Magnification of photomicrographs: 100×. (**J**) Correlation between *HMGA2* mRNA and protein expression. Statistical analyses were performed based on a confidence interval of 95%. Data with *p* < 0.05 were considered statistically significant. * = *p* < 0.05.

In order to investigate *HMGA2* gene expression profile in EEC, its mRNA levels were assessed by RT-qPCR. The results shown in Figure 3A reveal that *HMGA2* expression was positive in all 46 cases of EEC and normal tissue samples evaluated, and, although no statistically significance was observed between the different groups, *HMGA2* mRNA median expression values of all different EEC stages evaluated were higher than that of normal endometrial samples. In particular, an evident increase in *HMGA2* expression was observed in poorly differentiated tumors (G3) (*p* = 0.0268) when compared to normal endometrial samples (Figure 3B). However, differently from *HMGA1* gene expression, there was no statistically significant association between *HMGA2* gene expression and tumor staging (*p* = 0.0654) (Figure 3A) nor tumor size (Figure 3C). Association analysis between *HMGA2* expression data and patients’ clinicopathological parameters was performed, and no significant association was detected (Table S4). *HMGA2* expression levels were not associated with patients’ age either (r = 0.1512, *p* = 0.3392, data not shown).

Regarding HMGA2 protein expression, IHC was performed in the same six normal endometrial tissues, five EEC samples from stage IA, five from stage IB, five from stage II, five from stage III and five samples from stage IV analyzed for gene expression in order to verify whether protein expression correlates with mRNA expression. The 26 EEC samples assessed by immunohistochemistry were chosen randomly out of the total samples investigated for mRNA expression. HMGA2 staining was not detected in normal endometrial samples, whereas all EEC samples were positive, nevertheless, the intensity of the staining was weak and no correlation between late tumor stages and increased intensity was observed (Figure 3D–I and Supplementary Table S5). Moreover, there was no significant correlation between HMGA2 gene and protein expression (Figure 3J).

### 3.5. Reanalysis of TCGA Data Confirms HMGA1 Upregulation in EEC and Reveals Its Impact on Patients’ Survival

**Figure 4:**
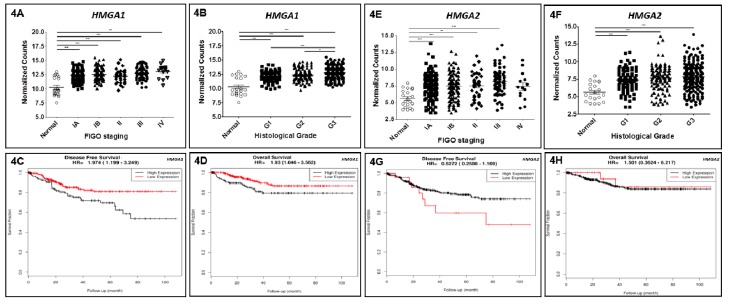
*HMGA1* and *HMGA2* expression in endometrioid endometrial adenocarcinomas (EEC) from The Cancer Genome Atlas (TCGA) cohort, and their potential as a prognostic biomarker. *HMGA1* and *HMGA2* gene expression reanalysis in EEC patients comprising TCGA cohort, according to clinicopathological characteristics. (**A**,**E**) Tumor staging: *HMGA1* and *HMGA2* expression was assessed in 139 EEC samples from stage IA, 123 from stage IB, 35 from stage II, 70 from stage III, 14 samples from stage IV and in 20 non-cancerous, tumor-adjacent endometrial tissue samples. (**B**,**F**) Histological grade: *HMGA1* and *HMGA2* expression reanalyzed in 95 well differentiated (G1), 107 moderately differentiated (G2), 179 poorly differentiated (G3) EEC samples and in 20 non-cancerous, tumor-adjacent endometrial tissue samples. (**C**,**D**,**G**,**H**) Survival analysis. (**C**,**D**). Evaluation of the association between the expression of *HMGA1* on the 381 EEC patients’ disease-free survival (**C**) (HR = 1.974, CI = 1.199–3.249, *p* = 0.00749) and overall survival (**D**) (HR = 1.930, CI = 1.046–3.562, *p* = 0.03) by univariate analysis. (**G**,**H**) Evaluation of the association between the expression of *HMGA2* on the 381 EEC patients’ disease-free survival (**G**) (HR = 0.5272, CI = 0.2508–1.109, *p* = 0.889) and overall survival (**H**) (HR = 1.51, CI = 0.3624–6.217, *p* = 0.67) by univariate analysis. *HMGA1* low expression < 12.92, *HMGA1* high expression ≥ 12.92; *HMGA2* low expression < 7.68, *HMGA2* high expression ≥ 7.68.

Seeking to confirm the results achieved in a larger series, reanalysis of expression data from 381 EEC samples and 20 non-malignant tumor-adjacent mucosas available in TCGA database was performed. Among the 381 EEC samples investigated, 139 were classified as stage IA, 123 as stage IB, 35 as stage II, 70 as stage III, and 14 as stage IV. Regarding gene expression reanalysis of TCGA EEC cohort, similarly to the results obtained in our initial set of samples, *HMGA1* was significantly overexpressed in tumors from all stages when compared with the non-cancerous tumor-adjacent endometrial tissues (Figure 4A). Considering the histological grade of tumors, a statistically significant augmentation of *HMGA1* expression was found in well, moderately and poorly differentiated tumors (G1, G2 and G3, respectively) when compared with tumor-adjacent endometrial samples (Figure 4B). The increase of *HMGA1* expression followed the loss of histological differentiation, since G3 EEC tumors presented significant higher *HMGA1* mRNA levels when compared with G1 and G2 tumors (Figure 4B). Finally, since TCGA EEC cohort was large enough, survival analyses were performed, and they revealed a significant association between *HMGA1* expression levels and overall survival (*p* = 0.03; HR = 1.93) in univariate analysis as well as with disease-free survival in both univariate (*p* = 0.00749; HR = 1.974) and multivariate analysis (*p* = 0.00334; HR = 1.7849), demonstrating its role as an independent prognostic factor (Figure 4C,D and Table 2).

*HMGA2* expression was also increased in TCGA EEC tumors from stages IA to III (Figure 4E) and from all histological grades (Figure 4F). Nevertheless, contrarily to *HMGA1*, there was no increment in *HMGA2* expression levels according to histological grade. Different from *HMGA1* expression, survival analyses demonstrated that *HMGA2* expression is not associated with either overall or disease-free survival of EEC patients in both univariate and multivariate analyses (Figure 4G,H and Table 2).

Next, in order to further investigate whether the gradual increase in *HMGA1* expression along with tumor staging and its impact on EEC patients’ survival could be related with a gain in tumor invasiveness, correlations analyses between *HMGA1* and *HMGA2* expression levels and those of the metalloproteinases *MMP2* and *MMP9* were performed using TCGA EEC expression data. A significant positive correlation was observed between *HMGA1* and *MMP9* expression in late stages (III and IV) EEC tumors (rho = 0.38, *p* < 0.01), and between *HMGA2* and *MMP2* in early stages (I and II) tumors (rho = 0.21, *p* = 0.008) (Table 3), suggesting a differential association pattern between the invasiveness markers and *HMGA* genes.

## 4. Discussion

In this study, we have analyzed the expression profile of HMGA genes in two different EEC samples series in order to access the potential of *HMGA* expression as a biomarker and, additionally, gain some insight on its tumor biology, since it is already known that alterations in HMGA expression are crucial for cancer development and progression [30].

Here, we report HMGA1 gene and protein overexpression in EEC samples from stage IA to IV, suggesting that *HMGA1* overexpression may play a role in EEC development and progression. In fact, Tesfaye et al. showed that HMGA1 plays an important role in the genesis of uterine tumors, since the targeted expression of HMGA1 to the uterine tissue was able to drive the development of tumors which resemble the uterine adenosarcoma in a mouse model [21]. Additionally, the same study demonstrated that the up-regulation of the inflammatory mediator COX-2 represents the signaling mechanism through which HMGA1 induces uterine neoplastic transformation.

Our results also demonstrate a significant upregulation of *HMGA1* expression following the increase in EEC histological tumor grade as well as a positive correlation between *HMGA1* expression and tumor size and an association between *HMGA1* levels and depth of myometrial invasion. Further, *HMGA1* expression was revealed as an independent prognostic factor for EEC patients’ disease-free survival. Thus, these results point out the role of *HMGA1* expression as a prognostic marker for EEC patients and demonstrate that its upregulation is related to a poorer outcome. In line with these observations, Hillion and colleagues recently evaluated *HMGA1* expression in three different histopathological types of uterine tumors (regardless of FIGO staging classification and histological grade within each of the groups investigated) and showed that HMGA1 is more expressed in high-grade uterine tumors, such as endometrial serous carcinoma and uterine carcinosarcoma, than in less aggressive ones, such as EEC, suggesting an increase in *HMGA1* expression along with uterine tumor aggressiveness [22]. Additionally, reanalysis of TCGA EEC cohort performed in our study revealed a significant positive correlation between *HMGA1* and *MMP9* expression levels only in late stage samples. MMP9 is a metalloproteinase from gelatinase class, which is classically involved with degradation of elements harbored within the basal membrane, such as collagens, a biological event highly associated with tumor invasion [30]. The correlation between *HMGA1* and *MMP9* expression in late stage EEC samples raises the hypothesis that the upregulation of HMGA1 may trigger tumor invasiveness through MMP9 upregulation and consequently lead to patients’ poorer prognosis. In fact, these data seem to be in accordance with previous studies that have shown the involvement of HMGA1 in the regulation of MMP9 expression along invasion and metastasis [31,32]. Finally, it has already been shown that HMGA1 overexpression is associated with patients’ poor outcome in several human neoplasias, such as colon carcinomas [33,34], pancreatic adenocarcinomas [35,36], non-small cell lung cancer [37] and breast tumors [38].

Further, the expression analysis of *HMGA1P6* and *HMGA1P7* pseudogenes showed the same pattern observed for *HMGA1* gene expression. Moreover, *HMGA1* levels were positively correlated with those of *HMGA1P6* and *HMGA1P7*, suggesting that these pseudogenes may participate in HMGA1 expression regulation. Indeed, it has been previously demonstrated that the overexpression of these pseudogenes leads to the increase in *HMGA1* levels by inhibiting driven suppression of *HMGA1* synthesis by microRNAs acting as competitive endogenous RNA. Thus, upregulation of *HMGA1P6* and *HMGA1P7* increases HMGA1 protein expression, representing a mechanism of its regulation [24]. In agreement with our results, *HMGA1P6* and *HMGA1P7* expression was also upregulated in anaplastic thyroid and ovarian carcinomas and pituitary tumors, where it significantly correlates with *HMGA1* overexpression [24,25].

Similar to the results observed for HMGA1 expression, *HMGA2* was also detected as overexpressed in EEC tumors. Nevertheless, *HMGA2* levels did not follow the increase in tumor histological grade, nor were they correlated with tumor size. Additionally, HMGA2 levels were not associated with EEC patients’ prognosis. The involvement of *HMGA2* in endometrioid endometrial carcinogenesis is controversial. Romero-Pérez and colleagues reported that HMGA2 mRNA and protein overexpression participates in nonendometrioid carcinomas and endometrial carcinosarcomas development but not in that of EEC, suggesting its levels as a potential marker to distinguish between endometrioid and nonendometrioid endometrial tumors [39]. Accordingly, Wei and colleagues showed that HMGA2 is highly expressed in endometrium serous carcinomas (about 90% of the samples evaluated were positively stained for HMGA2), whereas it is absent in 60% of the EEC samples assessed. Additionally, among EEC samples expressing HMGA2, protein levels were barely detected when compared with endometrium serous carcinoma or with endometrium benign lesions, such as glandular dysplasia and intraepithelial neoplasia [23]. Controversially, a recent study published by Ma and colleagues [40] also reported the involvement of HMGA2 in endometrial cancer development and progression, demonstrating increased expression levels of HMGA2 in late stages, high-grade, and more invasive tumors. Further, in this study, HMGA2 overexpression was shown to be associated with poorer overall survival of patients in univariate analysis. However, in this study, tumors were evaluated collectively as endometrial carcinomas and not stratified by histological subtype, which represent a large variation in prognosis. Finally, Montserrat et al. demonstrated that *HMGA2* is upregulated in EEC and that this phenomenon is associated with myometrial invasion [41]. HMGA2 upregulation and tissue invasion is a logical link, since HMGA2 is capable of regulating the epithelial-mesenchymal transition (EMT) process [42], and it could be one of the main mechanisms enabling invasion. However, although our results show *HMGA2* upregulation in EEC samples, they may not account for tumor invasion as we did not observe any correlation between *HMGA2* expression levels and those of the EMT regulators—*SNAIL*, *SLUG* and *TWIST* (data not shown). Additionally, there were no significant correlations between *HMGA2* and gelatinases—*MMP2* and *MMP9*—expression in late stage EEC samples. Therefore, more studies should be performed in order to further determine the role, if any, of HMGA2 in initiation and/or progression of endometrioid endometrial carcinogenesis.

HMGA1 and HMGA2 possess a highly similar structure and expression pattern. Nonetheless, studies using *HMGA1* and *HMGA2* null mice reported different phenotypes, suggesting that they have different functions [19]. In addition, a recent review proposed the differential induction of the HMGA proteins, depending on the cancer histological type, the induction of HMGA1 being an event usually observed in tumors originating from glandular tissues, whereas HMGA2 overexpression would participate in the development of tumors originating from epithelial cells [43]. This is in line with our findings showing that upregulation of HMGA1 but not of HMGA2 seems to participate in the initiation and/or progression of endometrioid endometrial carcinogenesis.

Together, these results point out HMGA1 overexpression as a potential prognostic biomarker for EEC.

## Figures and Tables

**Table 1:** Clinicopathological characteristics of the 46 endometrioid endometrial carcinoma (EEC) patients and six controls in the study.

Characteristics	Patients (%)	Controls (%)	*p*
Median age (years)	64	39.5	0.003
Variation	42–83	35–72	
Hypertension			0.07
Yes	29 (63.0)	1 (16.7)	
No	17 (37.0)	5 (83.3)	
Diabetes			0.57
Yes	9 (19.6)	0 (0)	
No	37 (80.4)	6 (100)	
Heart diseases			1.00
Yes	4 (8.7)	0 (0)	
No	42 (91.3)	6 (100)	
Obesity or Overweight			0.63
Yes	35 (76.1)	4 (66.7)	
No	11 (23.9)	2 (33.3)	
Nulliparity			1.00
Yes	6 (13.0)	0 (0)	
No	36 (78.3)	6 (100)	
N/A	4 (8.7)		
Contraceptive use			0.33
Yes	11 (23.9)	3 (50)	
No	32 (69.6)	3 (50)	
N/A	3 (6.5)	-	
Exogenous Estrogen Therapy			0.16
Yes	0 (0.0)	1 (16.7)	
No	42 (91.3)	5 (83.3)	
N/A	4 (8.7)	-	
Menopause			
Yes	38 (82.6)	3 (50.0)	0.06
No	6 (13.0)	3 (50.0)	
N/A	2 (4.4)	-	
FIGO Stage			
IA	10 (21.7)		
IB	10 (21.7)		
II	10 (21.7)		
III	10 (21.7)		
IV	6 (13.0)		
Histological Grade			
Well differentiated (G1)	11 (23.9)		
Moderate differentiated (G2)	22(47.8)		
Poorly differentiated (G3)	13 (28.3)		
Lymphovascular Infiltration			
Yes	7 (15.2)		
No	29 (63.0)		
N/A	10 (21.8)	-	
Myometrial Invasion			
<50%	22 (47.8)		
≥50%	24 (52.2)		
Recurrence			
Yes	13 (28.3)		
No	33 (71.7)		

Fisher’s exact test. N/A: Not informed.

**Table 2:** Univariate and multivariate overall and disease-free survival analyses of the 381 endometrioid endometrial adenocarcinoma (EEC) patients from The Cancer Genome Atlas (TCGA) database evaluated in the study.

**Overall Survival**				
	**Category**	**HR**	**95% CI**	***p* Value**
**Univariate Analysis**				
Age	<median vs. ≥median	1.872	0.9818–3.571	0.0569
Histological Grade	G3 vs. G2 vs. G1	2.381	1.457–3.892	**0.00054**
FIGO Stage	Late vs. Early	3.713	2.011–6.853	**0.0000274**
*HMGA1*	High vs. Low	1.93	1.046–3.562	**0.03**
*HMGA2*	High vs. Low	1.501	0.3624–6.217	0.67
**Multivariate Analysis**				
Age	<median vs. ≥median	2.03	1.0417–3.940	**0.03749**
Histological Grade	G3 vs. G2 vs. G1	2.013	1.2120–3.334	**0.00683**
FIGO Stage	Late vs. Early	3.33	1.7751–6.259	**0.00018**
*HMGA1*	High vs. Low	1,315	0.7192–2.558	0.34638
**Disease-Free Survival**				
	**Category**	**HR**	**95% CI**	***p* Value**
**Univariate Analysis**				
Age	<median vs. ≥median	1.628	0.9766–2.715	0.0616
Histological Grade	G3 vs. G2 vs. G1	1,453	1.054–2.003	**0.0224**
FIGO Stage	Late vs. Early	1.922	1.128–3.275	**0.0163**
*HMGA1*	High vs. Low	1.974	1.199–3.249	**0.00749**
*HMGA2*	High vs. Low	0.5272	0.2508–1.109	0.889
**Multivariate Analysis**				
Age	<median vs. ≥median	1.5992	0.9458–2.704	0.0798
Histological Grade	G3 vs. G2 vs. G1	1.2960	0.9298–1.806	0.1259
FIGO Stage	Late vs. Early	1.8945	1.0702–3.196	**0.0276**
*HMGA1*	High vs. Low	1.7849	1.0465–3.044	**0.0334**

Kaplan–Meier method and log-rank test. For multivariate analysis, Cox regression with the stepwise forward method. Multivariate analyses were adjusted by HMGA2 mRNA expression.

**Table 3:** Correlation analyses between *HMGA* genes and *metalloproteinases* (*MMP*) expression assessed in samples from 381 endometrioid endometrial adenocarcinoma (EEC) patients from The Cancer Genome Atlas (TCGA) database evaluated in the study.

	*HMGA1*				*HMGA2*			
	Early Stages		Late Stages		Early Stages		Late Stages	
	rho	padj	rho	padj	rho	padj	rho	padj
***MMP2***	0.13	0.6	0.12	1	**0.21**	**0.008**	0.33	0.07
***MMP9***	0.17	0.06	**0.38**	**<0.01**	0.12	0.8	0.003	1

## Supplementary Materials

The following are available online at https://www.mdpi.com/2073-4425/10/5/372/s1, Figure S1: Survival analysis of the 46 endometrioid endometrial adenocarcinoma (EEC) patients analyzed in this study, according to tumor staging, Table S1: Sequences of the five pairs of oligonucleotides sequences used in the study, Table S2: Associations between *HMGA1* gene expression and baseline characteristics of the 46 endometrioid endometrial adenocarcinoma (EEC) patients in the study, Table S3: HMGA1 protein expression by immunohistochemistry in 26 endometrioid endometrial carcinomas (EEC) patients in the study, Table S4: Associations between *HMGA2* gene expression and baseline characteristics of the 46 endometrioid endometrial adenocarcinoma (EEC) patients in the study, Table S5: HMGA2 protein expression by immunohistochemistry in 26 endometrioid endometrial carcinomas (EEC) patients in the study.
