# Supplementary material for: Overexpression of HMGA1 Figures as a Potential Prognostic Factor in Endometrioid Endometrial Carcinoma (EEC)
Source: Genes (Basel). 2019 May 15;10(5):372. doi: 10.3390/genes10050372 (PMC6562754; doi:10.3390/genes10050372)
Supplement: Supplementary file 1 [file genes-10-00372-s001.pdf]

1A

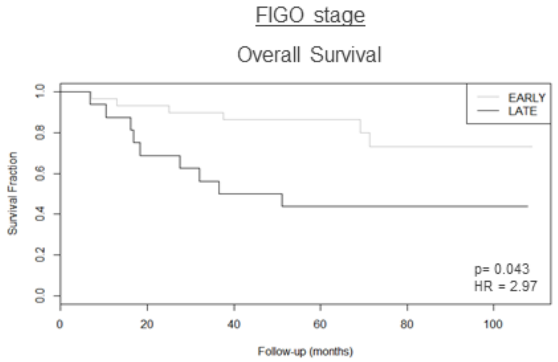


1B

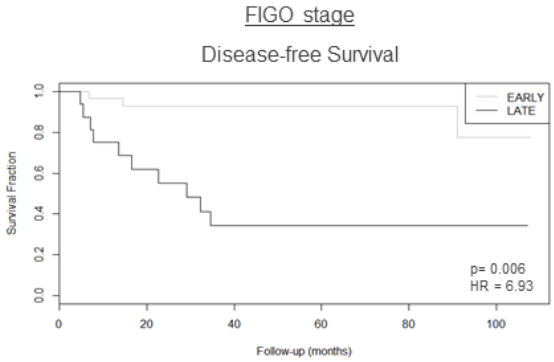


**Figure S1.** The Kaplan–Meier method was used to evaluate overall survival (1A) and disease-free survival (1B), based on a confidence interval of 95%. Data with $p < 0.05$ were considered statistically significant. Survival data of patients were plotted according to tumor stage: IA, IB and II were considered early stages; III and IV were considered late stages. Data were adjusted by stage and *HMGA1* expression in overall survival analysis and by stage, age and histological grade in disease-free survival analysis.

**Table S1.** Sequences of the five pairs of oligonucleotides sequences used in the study.

| **Oligonucleotides sequences** |
| --- |
| ***GAPDH*** |
| Forward:  5' CAACAGCCTCAAGATCATCAGCAA 3' |
| Reverse:  5' AGTGATGGCATGGACTGTGGTCAT 3' |
| ***HMGA1*** |
| Forward: 5' AAAAGGACGGCACTGAGAAG 3' |
| Reverse: 5' CTCTTAGGTGTTGGCACTTCG 3' |
| ***HMGA2*** |
| Forward: 5' GCGCCTCAGAAGAGAGGAC 3' |
| Reverse: 5' GGTCTCTTAGGAGAGGGCTCA 3' |
| ***HMGA1P6*** |
| Forward: 5'GCAGACCCACAAAACTGGA 3' |
| Reverse: 5' GAGCAAAGCTGTCCCATCC |
| ***HMGA1P7*** |
| Forward: 5' GCTCCTTCTCGGCTCCTC 3' |
| Reverse: 5' GCTTGGGCCTCTTTTATGG 3' |

**Table S2**: Correlations between *HMGA1* gene expression and baseline characteristics of patients.

| Characteristics | ≤ 0.089726* | >0.089726* | *p* value |
|---|---|---|---|
| **Hypertension** | | | |
| **Yes** | 14 (48.3%) | 15 (51.7%) | 1.0000 |
| **No** | 9 (52.9%) | 8 (47.1%) | |
| **Diabetes** | | | |
| **Yes** | 6 (66.7%) | 3 (33.3%) | 0.4591 |
| **No** | 17 (45.9%) | 20 (54.1%) | |
| **Heart diseases** | | | |
| **Yes** | 1 (25.0%) | 3 (75.0%) | 0.6076 |
| **No** | 22 (52.4%) | 20 (47.6%) | |
| **Obesity or Overweight** | | | |
| **Yes** | 18 (51.4%) | 17 (48.6%) | 1.0000 |
| **No** | 5 (45.5%) | 6 (54.5%) | |
| **Nulliparity** | | | |
| **Yes** | 4 (66.7%) | 2 (33.3%) | 0.6628 |
| **No** | 17 (47.2%) | 19 (52.8%) | |
| **Contraceptive use** | | | |
| **Yes** | 6 (54.5%) | 5 (45.5%) | 1.0000 |
| **No** | 16 (50.0%) | 16 (50.0%) | |
| **Histological Grade** | | | |
| **G1** | 6 (54.5%) | 5(45.5%) | |
| **G2** | 13 (59.1%) | 9 (40.9%) | 0.2539 |
| **G3** | 4 (30.8%) | 9 (69.2%) | |
| **Recurrence** | | | |
| **Yes** | 6 (46.2%) | 7 (53.8%) | 1.0000 |
| **No** | 17 (51.5%) | 16 (48.5%) | |
| **Myometrial Invasion** | | | |
| **≥ 50%** | 8 (33.3%) | 16 (66.7%) | **0.0377** |
| **< 50%** | 15 (68.2%) | 7 (31.8%) | |
| **Lymphovascular Infiltration** | | | |
| **Yes** | 3 (42.9%) | 4 (57.2%) | 0.6843 |
| **No** | 16(55.2%) | 13(44.8%) | |

*Fisher's exact test*

** Patients were divided in two groups, according to HMGA1 mRNA median expression levels (0.089726).*

**Table S3:** HMGA1 protein expression by immunohistochemistry in 26 endometrioid endometrial carcinomas (EEC) samples.

| Histological Type | HMGA1 Staining Score (n) | | | | |
|---|---|---|---|---|---|
| | 0 | 1+ | 2+ | 3+ | 4+ |
| EEC Stage IA (n=5) | 0 | 3 | 1 | 1 | 0 |
| EEC Stage IB (n=5) | 0 | 0 | 1 | 3 | 1 |
| EEC Stage II (n=5) | 0 | 0 | 2 | 3 | 0 |
| EEC Stage III (n=5) | 0 | 0 | 1 | 2 | 2 |
| EEC Stage IV (n=6) | 0 | 0 | 1 | 1 | 4 |

**Table S4:** Correlations between *HMGA2* gene expression and baseline characteristics of patients.

| Characteristics | ≤ 0.008599* | >0.008599* | *p* value |
|---|---|---|---|
| **Hypertension** | | | |
| **Yes** | 16 (55.2%) | 13 (44.8%) | 0.5422 |
| **No** | 7 (41.2%) | 10 (58.8%) | |
| **Diabetes** | | | |
| **Yes** | 5 (55.6%) | 4 (44.4%) | 1.0000 |
| **No** | 18 (48.6%) | 19 (51.4%) | |
| **Heart diseases** | | | |
| **Yes** | 3 (75%) | 1 (25%) | 0.6078 |
| **No** | 20 (47.6%) | 22 (52.4%) | |
| **Obesity or Overweight** | | | |
| **Yes** | 18 (51.4%) | 17 (48.6%) | 0.4966 |
| **No** | 4 (36.4%) | 7 (63.6%) | |
| **Nulliparity** | | | |
| **Yes** | 4 (66.7%) | 2 (33.3%) | 0.1249 |
| **No** | 18 (50%) | 18 (50%) | |
| **Contraceptive use** | | | |
| **Yes** | 4 (36.4%) | 7 (63.6%) | 0.3102 |
| **No** | 18 (56.3%) | 14 (43.7%) | |
| **Histological grade** | | | |
| **G1** | 5 (45.5%) | 6 (54.5%) | 0.4699 |
| **G2** | 13 (59.1%) | 9 (40.9%) | |
| **G3** | 5 (38.5%) | 8 (61.5%) | |
| **Recurrence** | | | |
| **Yes** | 5 (38.5%) | 8 (61.5%) | 0.3141 |
| **No** | 19 (55.9%) | 15 (44.1%) | |
| **Myometrial Invasion** | | | |
| **≥ 50%** | 14 (58.3%) | 10 (41.7%) | 0.3762 |
| **< 50%** | 9 (40.9%) | 13 (59.1%) | |
| **Lymphovascular Infiltration** | | | |
| **Yes** | 3 (42.9%) | 4 (57.1%) | 0.4178 |
| **No** | 18 (62.1%) | 11 (37.9%) | |

*Fisher's exact test*

** Patients were divided in two groups, according to HMGA2 mRNA median expression levels (0.008599).*

**Table S5:** HMGA2 protein expression by immunohistochemistry in 26 endometrioid endometrial carcinomas (EEC) samples.

| | HMGA2 Staining Score (n) | | | | |
|---|---|---|---|---|---|
| **Histological Type** | 0 | 1+ | 2+ | 3+ | 4+ |
| EEC Stage IA (n=5) | 0 | 3 | 1 | 1 | 0 |
| EEC Stage IB (n=5) | 0 | 0 | 1 | 2 | 2 |
| EEC Stage II (n=5) | 0 | 0 | 2 | 3 | 0 |
| EEC Stage III (n=5) | 0 | 0 | 1 | 3 | 1 |
| EEC Stage IV (n=6) | 0 | 0 | 1 | 3 | 2 |
